# Incidence of Metabolic Syndrome in Young Japanese Adults in a 6-Year Cohort Study: The Uguisudani Preventive Health Large-Scale Cohort Study (UPHLS)

**DOI:** 10.2188/jea.JE20180246

**Published:** 2020-05-05

**Authors:** Yasuo Haruyama, Ayako Nakagawa, Kumiko Kato, Masayo Motoi, Toshimi Sairenchi, Mitsumasa Umesawa, Ayako Uematsu, Yuichirou Kudou, Gen Kobashi

**Affiliations:** 1 Department of Public Health, Dokkyo Medical University School of Medicine, Tochigi, Japan; 2 Department of Medicine, Uguisudani Medical Center, Tokyo, Japan

**Keywords:** metabolic syndrome, incidence, young adult, cohort study, epidemiology

## Abstract

**Introduction:**

To clarify the incidences of metabolic syndrome (MS) and risks in young Japanese adults by gender.

**Methods:**

A total of 58,901 adults who had undergone annual health check-ups in 2010 without a diagnosis of MS or missing data were divided into three age groups (20s through 40s) by gender. Participants were followed up for 6 years for new-onset MS according to Japanese criteria. The incidences of MS and risks were analyzed using the Cox proportional hazards model to adjust for confounding factors.

**Results:**

The incidences of MS per 1,000 person-years were 2.2, 5.5, and 10.2 for women aged in their 20s, 30s, and 40s, respectively, and 26.3, 40.5, and 57.4 in the respective men groups. Compared with the group aged in their 40s, the hazard ratios of new MS were 0.19 (95% confidence interval [CI], 0.13–0.29) for women in their 20s and 0.50 (95% CI, 0.41–0.61) for women in their 30s, and 0.46 (95% CI, 0.42–0.50) and 0.70 (95% CI, 0.66–0.73) for men in their 20s and 30s, respectively, after adjustment for lifestyle factors. For women, MS was associated with smoking in their 20s and 30s, and eating speed in their 30s, and for men, was associated with physical activity, eating speed, alcohol intake in their 20s and 30s, and smoking in their 30s.

**Conclusion:**

Our findings suggest that the incidences of MS in the 20s and 30s are lower, but account for about 20–50% of women with MS and 50–70% of men with MS in their 40s. However, the data are not negligible and early lifestyle intervention for MS is necessary in young adults.

## INTRODUCTION

Metabolic syndrome (MS) is a clustering of risks for cardiovascular diseases in middle-aged adults, such as ischemic heart disease and stroke, seen in populations worldwide.^1^^–^^3^ Many prior studies reported different incidences in MS from 3.1 to 22.9 per 1,000 person-years using different definition criteria for adults aged 40 years or older.^4^^–^^7^ Regarding the incidence of MS per 1,000 person-years in young adults aged younger than 40, values of 11.7 for men and women aged 18–30 years^8^; 13.3 for men and women aged 20–32 years^9^; 12.0 for women aged 18–30 years^10^; and 20.4 for black men and 24.9 for white men, and 27.9 for black women and 12.5 for white women aged 18–30 years^11^ were reported in the United States using NCEP-ATP III criteria. In addition, Dreyfus et al reported a cumulative incidence of 24.8% in 2,534 African-American and white women aged 18–30 years during a 25-year follow-up.^12^ However, in Asians, some studies^13^^,^^14^ discussed the association between MS incidence and subject factors including ages 20–30 years, but the incidence of MS in young people is unclear. Also, in Japan, one retrospective study reported a MS incidence of 9.2% in 877 men workers aged 30–35 according to Japanese criteria using BMI.^15^ Prospective studies reported an incidence rate of MS per 1,000 person-years of 24.0 in 22,383 workers aged 30–64 years,^16^ and a 12% cumulative incidence rate of MS in 6,817 workers aged 20 years or older during a median follow-up of 3 years defined by the Joint Statement criteria.^17^

Although these previous studies included those in their 20s or 30s, they did not focus on the incidence rate of MS in young Japanese adults according to Japanese criteria. Further, to our knowledge, no published large-scale prospective cohort study has analyzed the incidence rate of MS in young Japanese adults in their 20s to 30s by gender according to Japanese criteria.

In 2008, a national health program was launched in Japan. This program is called specific health checkups (SHC) and specific health guidance (SHG), focusing on improving the status of MS.^18^ The targets of this program are people from 40 to 74 years old with MS or pre-MS. However, after the first term (2008–2013) and second term (2013–2018) of this national program, the prevalence of MS in Japanese people aged 40 years or older is still high.^19^ We hypothesized that many people younger than 40 years developed new MS because they are not a target population. Hence, the aim of the present study was to clarify the incidence of MS among young Japanese adults by gender in a large-scale 6-year prospective cohort study.

## METHODS

### Study design and participants

**Figure 1.**
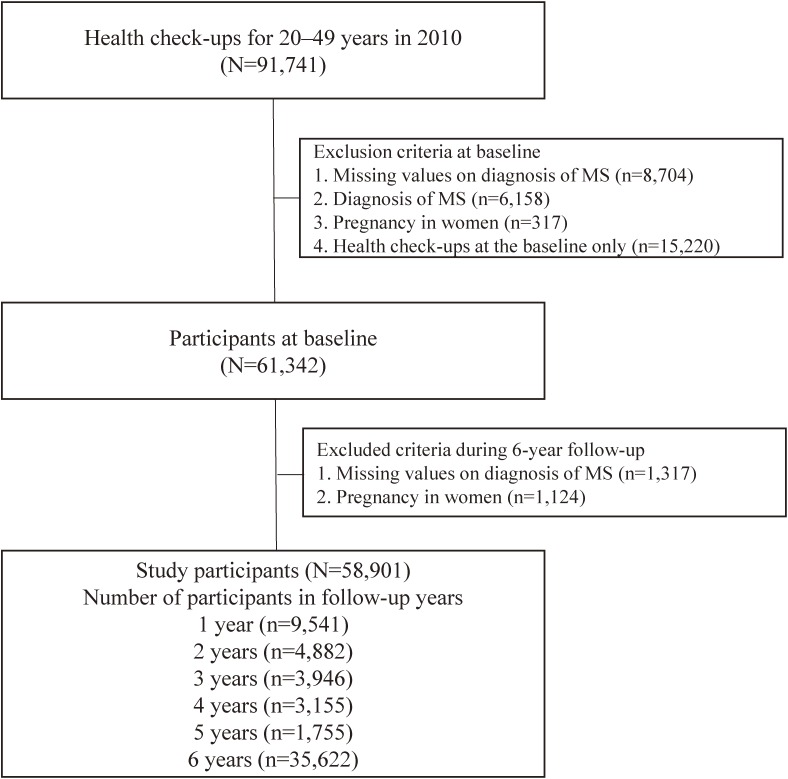
Flow chart of the study

A 6-year follow-up cohort study was conducted. Subjects were recruited from participants who underwent annual health checkups from 2010 to 2016 in the Uguisudani Medical Center in Tokyo, Japan. In 2010, 56,897 participants were workers aged from 20 to 39 years old who underwent regular health checkups according to the Industrial Safety and Health Law, and 34,844 participants aged from 40 to 49 years old were from specific health checkups according to the Law Concerning the Security of Healthcare Treatment for Senior Citizens in Japan. Of the 91,741 participants, those with missing data regarding the diagnosis of MS (*n* = 8,704), history of MS diagnosis (*n* = 6,158), pregnancy in women (*n* = 317), and those who had undergone health check-ups at the baseline only (*n* = 15,220) were excluded. A final target of 61,342 participants without a diagnosis of MS participated in the baseline survey. We excluded 1,317 participants because they had missing data on the diagnosis of MS and 1,124 pregnancies during the 6-year follow-up period. Therefore, 58,901 participants were enrolled in the present study and followed until 2016. Of the 58,901 participants, 35,622 (60.5%) were followed for 6 years, and the other 23,279 (39.5%) participants as censored cases did not visit the center continuously from the third to fifth years after the follow-up of this study (Figure 1).

### Measurements

The baseline and follow-up annual health checkups included a physical examination, anthropometric measurements, a medical questionnaire (including the history of present and past illnesses), and a medication history. All of the measurements were provided by Uguisudani Medical Center. Waist circumference (WC) was measured by nurses. Systolic blood pressure (SBP) and diastolic blood pressure (DBP) were measured using auto-manometers (Omron Co., Tokyo, Japan). Fasting blood samples were obtained from all subjects, and triglycerides (TG), low-density lipoprotein cholesterol (LDL-C), high-density lipoprotein cholesterol (HDL-C), and fasting blood glucose (FBG) were measured at a biomedical laboratory (BML, Inc., Tokyo, Japan). The medical history included questions regarding the history of hypertension or taking hypertension drugs, history of dyslipidemia or taking lipid-lowering drugs, and history of diabetes or taking diabetes medication.

Lifestyle factors, such as smoking, drinking alcohol, dietary behaviors, physical activity, and sleeping condition were obtained via a medical questionnaire at baseline. The smoking status was classified as current smoker, ex-smoker, and non-smoker. Drinking alcohol was classified by the frequency of drinking per week and month and the amount of alcohol consumed per day. Dietary behaviors included eating speed and frequency of eating dinner late, eating snacks, and skipping breakfast. Physical activity included regular exercise and daily physical activity.

### Outcome assessment of metabolic syndrome

Metabolic syndrome was diagnosed according to Japanese criteria from the Japanese Society of Internal Medicine^20^ and National Cholesterol Education Program Adult Treatment Panel III (NCEP-ATP III).^21^

Japanese criteria are based on the following inclusion criteria^20^: a) WC ≥85 cm for men and ≥90 cm for women are essential conditions, plus two or more of the following three criteria: b) FBG >110 mg/dL or taking diabetes medication; c) TG >150 mg/dL or HDL-C <40 mg/dL or taking lipid-lowering medication; and d) SBP ≥130 mm Hg or DBP ≥85 mm Hg or taking hypertension drugs.

Using NCEP-ATP III criteria,^21^ a participant was diagnosed with MS when three or more of the following five criteria were satisfied: a) WC >102 cm for men or >88 cm for women; b) HDL-C <40 mg/dL in men or <50 mg/dL in women; c) TG ≥150 mg/dL; d) SBP >130 mm Hg or DBP >85 mm Hg or taking hypertension medication; and e) FBG ≥110 mg/dL.

In the present study, the period diagnosed with metabolic syndrome was counted from the year in which the health check-up had been undertaken. When a health check-up was not undergone for the first year after the baseline, it was considered a censored case.

### Statistical analyses

The participants were divided into three age groups (20s, 30s, and 40s at baseline) by gender. Baseline characteristics were analyzed using the Chi-squared test for categorical variables of the components of MS and lifestyles among age groups. Person-years were calculated as the sum of individual follow-up times until the occurrence of new metabolic syndrome, censored cases, or the end of the follow-up in 2016. The incidence of MS among age groups by gender was evaluated using Cox proportional hazards models adjusted for multiple potential confounding factors (smoking, drinking alcohol, dietary behavior, physical activity, sleeping, and medication history). The incidence of MS among people who did not fulfill any components of MS at the baseline was also analyzed. The lifestyle, as a risk factor of new-onset MS among age groups, was analyzed by gender. Alcohol consumption was entered only because the correlation coefficient was 0.636 between alcohol frequency and consumption. All statistical analyses were performed using an assumed type I error rate of 0.05. Statistical analyses were performed using SPSS Statistics 24 for Windows (IBM Japan, Tokyo, Japan).

### Ethical consideration

This cohort study was conducted in compliance with the ethical guidelines on medical research for people of the Ministry of Education, Culture, Sports, Science and Technology and the Ministry of Health, Labour and Welfare of Japan,^22^ and with the personal information protections of Uguisudani Medical Center. Ethics approval of this study protocol was given by the ethics committee of Dokkyo Medical University (No. 24004).

## RESULTS

### Baseline characteristics

There were 17,665 women and the mean age was 37.3 (standard deviation [SD], 7.5) years: 3,458 (19.6%) in their 20s, 6,395 (36.2%) in their 30s, and 7,812 (44.2%) in their 40s without current MS or a history of MS according to the Japanese criteria. Except for sleeping condition, the MS components, medication, and other lifestyle factors showed significant differences among the three women age groups shown in Table 1. For men, there were 41,236 people and the mean age was 37.4 (SD, 6.8) years: 6,241 (15.1%) in their 20s, 17,946 (43.5%) in their 30s, and 17,049 (41.3%) in their 40s. Table 2 shows that there were significant differences in all items except for the sleeping condition.

**Table 1.** Characteristics of women participants at baseline

	20–29 years		30–39 years		40–49 years		*P* value^a^
	(*n* = 3,458)		(*n* = 6,395)		(*n* = 7,812)		
		
	*n*	%	*n*	%	*n*	%	
*Health check-up*							
Abdominal circumference, ≥90 cm	127	3.7	398	6.2	659	8.4	<0.001
Systolic blood pressure, ≥130 mm Hg	93	2.7	293	4.6	1,065	13.6	<0.001
Diastolic blood pressure, ≥85 mm Hg	39	1.1	255	4.0	775	9.9	<0.001
Triglycerides, ≥150 mg/dL	45	1.3	153	2.4	347	4.4	<0.001
HDL-cholesterol, <40 mg/dL	12	0.3	63	1.0	76	1.0	0.001
Fasting blood glucose, ≥110 mg/dL	18	0.5	62	1.0	212	2.7	<0.001
*Medication*							
Taking hypertension medication	0	0.00	20	0.31	182	2.33	<0.001
Taking diabetes medication	1	0.03	9	0.14	36	0.46	<0.001
Taking lipid-lowering medication	4	0.12	5	0.08	62	0.79	<0.001
*Lifestyles*							
Smoking condition^b^							
Non-smoker	2,653	76.7	4,202	65.7	5,138	65.8	<0.001
Current smoker	508	14.7	1,226	19.2	1,543	19.8	
Ex-smoker	297	8.6	966	15.1	1,129	14.5	
Frequency of drinking alcohol^b^							
No drinking	656	19.0	1,657	25.9	2,396	30.7	<0.001
1–2 day per month	1,564	45.2	2,029	31.8	1,950	25.0	
1–2 day per week	915	26.5	1,339	21.0	1,307	16.7	
3–4 day per week	191	5.5	614	9.6	765	9.8	
More than 5 days per week	131	3.8	751	11.8	1,390	17.8	
Alcohol intake^b^							
No drinking	653	18.9	1,652	25.9	2,395	30.7	<0.001
Less than 20 g per day	1,549	44.9	2,379	37.3	2,813	36.1	
20–60 g per day	1,134	32.9	2,179	34.2	2,419	31.0	
More than 60 g per day	112	3.2	166	2.6	174	2.2	
30 minutes of exercise a week at least twice per week^b^	294	8.5	853	13.3	1,203	15.4	<0.001
Physical activity over hour per day^b^	1,341	38.8	2,009	31.4	2,827	36.2	<0.001
Eating speed							
Slow	726	21.0	1,007	15.8	907	11.6	<0.001
General	1,819	52.6	3,719	58.2	4,752	60.8	
Fast	912	26.4	1,666	26.1	2,151	27.5	
Eating dinner less than 2 hours before sleeping more than 3 times per week^b^	1,300	37.6	2,114	33.1	2,238	28.7	<0.001
Eating snacks after dinner more than 3 times per week^b^	679	19.6	1,119	17.5	1,405	18.0	0.029
Skipping breakfast more than 3 times per week^b^	1,180	34.1	1,770	27.7	1,669	21.4	<0.001
Sleeping well^b^	1,675	48.5	3,107	48.6	3,675	47.1	0.140

^a^Using a Chi-squared test.

^b^Missing values were excluded in smoking (*n* = 3), alcohol frequency (*n* = 10), alcohol intake (*n* = 40), exercise (*n* = 6), physical activity (*n* = 9), eating speed (*n* = 6), eating dinner less than 2 hours (*n* = 7), eating snacks (*n* = 4), skipping breakfast (*n* = 5), and sleeping (*n* = 6).

**Table 2.** Characteristics of men participants at baseline

	20–29 years		30–39 years		40–49 years		*P* value
	(*n* = 6,241)		(*n* = 17,946)		(*n* = 17,049)		
		
	*n*	%	*n*	%	*n*	%	
*Health check-up*							
Abdominal circumference, ≥90 cm	1,336	21.4	5,482	30.5	6,331	37.1	<0.001
Systolic blood pressure, ≥130 mm Hg	768	12.3	2,227	12.4	3,044	17.9	<0.001
Diastolic blood pressure, ≥85 mm Hg	338	5.4	1,607	9.0	2,764	16.2	<0.001
Triglycerides, ≥150 mg/dL	565	9.1	3,006	16.8	3,737	21.9	<0.001
HDL-cholesterol, <40 mg/dL	299	4.8	1,238	6.9	1,157	6.8	<0.001
Fasting blood glucose, ≥110 mg/dL	96	1.5	453	2.5	1,045	6.1	<0.001
*Medication*							
Taking hypertension medication	8	0.13	80	0.45	530	3.11	<0.001
Taking diabetes medication	4	0.06	25	0.14	120	0.70	<0.001
Taking lipid-lowering medication	2	0.03	58	0.32	248	1.45	<0.001
*Lifestyles*							
Smoking condition^b^							
Non-smoker	2,805	44.9	5,886	32.8	4,623	27.1	<0.001
Current smoker	2,706	43.4	8,297	46.2	7,845	46.0	
Ex-smoker	730	11.7	3,763	21.0	4,580	26.9	
Frequency of drinking alcohol^b^							
No drinking	989	15.8	2,970	16.6	2,774	16.3	<0.001
1–2 day per month	2,264	36.3	4,256	23.7	2,827	16.6	
1–2 day per week	1,716	27.5	3,830	21.3	2,855	16.7	
3–4 day per week	652	10.4	2,421	13.5	2,639	15.5	
More than 5 days per week	619	9.9	4,468	24.9	5,952	34.9	
Alcohol intake^b^							
No drinking	983	15.8	2,951	16.5	2,761	16.2	
Less than 20 g per day	2,016	32.4	5,325	29.7	4,555	26.8	
20–60 g per day	2,785	44.7	8,462	47.2	8,524	50.1	<0.001
More than 60 g per day	445	7.1	1,177	6.6	1,180	6.9	
30 minutes of exercise a week at least twice per week^b^	1,287	20.6	3,435	19.1	3,521	20.7	<0.001
Physical activity over hour per day^b^	3,324	53.3	7,888	44.0	6,644	39.0	<0.001
Eating speed							
Slow	804	12.9	1,675	9.3	1,430	8.4	<0.001
General	3,121	50.0	9,291	51.8	9,534	55.9	
Fast	2,316	37.1	6,978	38.9	6,083	35.7	
Eating dinner less than 2 hours before sleeping more than 3 times per week^b^	3,255	52.2	9,740	54.3	9,338	54.8	0.002
Eating snacks after dinner more than 3 times per week^b^	1,327	21.3	2,937	16.4	2,348	13.8	<0.001
Skipping breakfast more than 3 times per week^b^	3,024	48.5	7,605	42.4	5,485	32.2	<0.001
Sleeping well^b^	2,910	46.6	8,440	47.0	8,038	47.2	0.778

^a^Using a Chi-squared test.

^b^Missing values were excluded in smoking (*n* = 1), alcohol frequency (*n* = 4), alcohol (*n* = 72), exercise (*n* = 8), physical activity (*n* = 9), eating speed (*n* = 4), eating dinner less than 2 hours (*n* = 8), eating snacks (*n* = 9), skipping breakfast (*n* = 13), and sleeping (*n* = 6).

### New-onset MS and age groups

A total of 7,828 new MS cases (496 women and 7,332 men) were diagnosed according to Japanese criteria and 229,019 person-years were observed during the 6-year follow-up. Table 3 shows the associations between age groups and new MS. The incidences of MS per 1,000 person-years were 2.2 for women in their 20’s, 5.5 for women in their 30s, and 10.2 for women in their 40s, and 26.3 for men in their 20s, 40.5 for men in their 30s, and 57.4 for men in their 40s.

**Table 3.** Associations between age groups and new MS during 6-year follow-up

	Age groups			*P* for trend

	20–29 years	30–39 years	40–49 years	
*Japanese criteria^a^ for MS*				
Women, *n*	3,458	6,395	7,812	
New MS, *n* (%)	25 (0.7)	132 (2.1)	339 (4.3)	
Person-years	11,302	23,819	33,169	
Incidence of MS per 1,000 person-years	2.2	5.5	10.2	
Crude hazard ratios	0.22	0.54	1.00	<0.001
95% CI	0.14–0.32	0.44–0.66		
Adjusted hazard ratios^c^	0.19	0.50	1.00	<0.001
95% CI	0.13–0.29	0.41–0.61		
Men, *n*	6,241	17,946	17,049	
New MS, *n* (%)	611 (9.8)	2,796 (15.6)	3,925 (23.0)	
Person-years	23,211	69,086	68,432	
Incidence of MS per 1,000 person-years	26.3	40.5	57.4	
Crude hazard ratios	0.46	0.71	1.00	<0.001
95% CI	0.42–0.50	0.67–0.74		
Adjusted hazard ratios^c^	0.46	0.70	1.00	<0.001
95% CI	0.42–0.50	0.66–0.73		

*NCEP-ATP II^b^ for MS*				
Women, *n*	4,693	7,272	7,941	
New MS, *n* (%)	83 (1.8)	298 (4.1)	563 (7.1)	
Person-years	13,569	23,942	29,101	
Incidence of MS per 1,000 person-years	6.1	12.5	19.3	
Crude hazard ratios	0.32	0.65	1.00	<0.001
95% CI	0.25–0.40	0.56–0.75		
Adjusted hazard ratios^c^	0.29	0.61	1.00	<0.001
95% CI	0.23–0.37	0.52–0.70		
Men, *n*	8,661	20,447	18,849	
New MS, *n* (%)	387 (4.5)	1,789 (8.7)	2,517 (13.1)	
Person-years	31,614	84,572	79,922	
Incidence of MS per 1,000 person-years	12.4	21.2	31.4	
Crude hazard ratios	0.38	0.67	1.00	<0.001
95% CI	0.34–0.42	0.63–0.71		
Adjusted hazard ratios^c^	0.36	0.64	1.00	<0.001
95% CI	0.32–0.40	0.60–0.68		

CI, confidence interval; MS, metabolic syndrome; NCEP-ATP, National Cholesterol Education Program Adult Treatment Panel III.

^a^Waist circumference ≥85 cm for men and ≥90 cm for women are essential conditions; and plus two or more of the following three criteria: 1) fasting blood glucose >110 mg/dL or taking diabetes medication; 2) triglycerides >150 mg/dL or high-density-lipoprotein cholesterol <40 mg/dL or taking lipid-lowering medication; 3) systolic blood pressure ≥130 mm Hg or diastolic blood pressure ≥85 mm Hg or taking hypertension drugs.

^b^When three or more of the following five criteria were satisfied: 1) Waist circumference >102 cm for men or >88 cm for women; 2) high-density-lipoprotein cholesterol <40 mg/dL in men or <50 mg/dL in women; 3) triglycerides ≥150 mg/dL; 4) systolic blood pressure >130 mm Hg or diastolic blood pressure >85 mm Hg or taking hypertension medication; 5) fasting blood glucose ≥110 mg/dL.

^c^Using a Cox proportional hazard model adjusted for smoking, alcohol intake, exercise habits, physical activity, eating speed, dinner late, snacking, and sleeping condition.

According to Japanese criteria, compared with those in their 40s, the fully adjusted multivariable hazard ratios adjusted for smoking, drinking alcohol, dietary behavior, physical activity, and sleeping were 0.50 (95% CI, 0.41–0.61) for women in their 30s and 0.19 (95% CI, 0.13–0.29) for women in their 20s, and 0.70 (95% CI, 0.66–0.73) for men in their 30s and 0.46 (95% CI, 0.42–0.50) for men in their 20s. The trend test was significant among the three age groups (all *P* < 0.001).

With NCEP-ATP III criteria, a total of 5,637 new MS cases (944 women and 4,693 men) were diagnosed, and person-years were 262,720. The incidences of MS per 1,000 person-years were 6.1 for women in their 20s, 12.5 for women in their 30s, and 19.3 for women in their 40s, and 12.4 for men in their 20s, 21.2 for men in their 30s, and 31.4 for men in their 40s.

According to NCEP-ATP III criteria, compared with those in their 40s, the fully multivariable adjusted hazard ratios adjusted for smoking, drinking alcohol, dietary behavior, physical activity, and sleeping were 0.61 (95% CI, 0.52–0.70) for women in their 30s, and 0.29 (95% CI, 0.23–0.37) for women in the 20s, and 0.64 (95% CI, 0.60–0.68) for men in their 30s and 0.36 (95% CI, 0.32–0.40) for men in their 20s. The trend test was significant among the three age groups (all *P* < 0.001).

### MS onset in healthy people

Table 4 shows that the incidences of MS in healthy people who did not exhibit any components of MS at the baseline were lower than in Table 3 during a 6-year follow-up. After adjustment for same confounding factors, compared with those in their 40s, the men in their 30s and 20s showed lower HR of MS onset: 0.78 (95% CI, 0.66–0.92) and 0.69 (95% CI, 0.54–0.88), respectively, according to Japanese criteria. According to NCEP-ATP III criteria, men and women in their 20s showed HRs of 0.65 (95% CI, 0.52–0.82) and 0.56 (95% CI, 0.37–0.86). The adjusted HR showed a significant tendency among the three age groups for men and women according to Japanese or NCEP-ATP III criteria.

**Table 4.** Associations between age groups without any component of MS and new MS during 6-year follow-up

	Age groups			*P* for trend

	20–29 years	30–39 years	40–49 years	
*Japanese criteria^a^ for MS*				
Women, *n*	3,193	5,528	5,702	
New MS, *n* (%)	7 (0.2)	22 (0.4)	43 (0.8)	
Person-years	10,409	20,713	24,887	
Incidence of MS, per 1,000 person-year	0.7	1.1	1.7	
Crude hazard ratios	0.44	0.65	1.00	0.018
95% CI	0.20–0.97	0.39–1.09		
Adjusted hazard ratios^c^	0.50	0.65	1.00	0.027
95% CI	0.22–1.14	0.38–1.09		
Men, *n*	3,904	9,027	6,259	
New MS, *n* (%)	97 (2.5)	291 (3.2)	293 (4.7)	
Person-years	15,045	37,587	28,411	
Incidence of MS, per 1,000 person-year	6.4	7.7	10.3	
Crude hazard ratios	0.65	0.77	1.00	<0.001
95% CI	0.52–0.82	0.66–0.91		
Adjusted hazard ratios^c^	0.69	0.78	1.00	<0.001
95% CI	0.54–0.88	0.66–0.92		

*NCEP-ATP III^b^ for MS*				
Women, *n*	4,188	7,970	5,571	
New MS, *n* (%)	29 (0.7)	71 (1.2)	99 (1.8)	
Person-years	12,069	19,886	21,122	
Incidence of MS, per 1,000 person-year	2.4	3.6	4.7	
Crude hazard ratios	0.59	0.81	1.00	0.009
95% CI	0.39–0.89	0.60–1.10		
Adjusted hazard ratios^c^	0.56	0.77	1.00	0.005
95% CI	0.37–0.86	0.56–1.05		
Men, *n*	6,397	12,722	8,917	
New MS, *n* (%)	110 (1.7)	336 (2.6)	277 (3.1)	
Person-years	23,476	54,711	40,545	
Incidence of MS, per 1,000 person-year	4.7	6.1	6.8	
Crude hazard ratios	0.71	0.91	1.00	0.003
95% CI	0.57–0.89	0.77–1.07		
Adjusted hazard ratios^c^	0.65	0.85	1.00	<0.001
95% CI	0.52–0.82	0.72–1.00		

CI, confidence interval; MS, metabolic syndrome; NCEP-ATP, National Cholesterol Education Program Adult Treatment Panel III.

^a^Waist circumference ≥85 cm for men and ≥90 cm for women are essential conditions; and plus two or more of the following three criteria: 1) fasting blood glucose >110 mg/dL or taking diabetes medication; 2) triglycerides >150 mg/dL or high-density-lipoprotein cholesterol <40 mg/dL or taking lipid-lowering medication; 3) systolic blood pressure ≥130 mm Hg or diastolic blood pressure ≥85 mm Hg or taking hypertension drugs.

^b^When three or more of the following five criteria were satisfied: 1) Waist circumference >102 cm for men or >88 cm for women; 2) high-density-lipoprotein cholesterol <40 mg/dL in men or <50 mg/dL in women; 3) triglycerides ≥150 mg/dL; 4) systolic blood pressure >130 mm Hg or diastolic blood pressure >85 mm Hg or taking hypertension medication; 5) fasting blood glucose ≥110 mg/dL.

^c^Using a Cox proportional hazard model adjusted for smoking, alcohol intake, exercise habits, physical activity, eating speed, dinner late, snacking, and sleeping condition.

### MS onset and lifestyles

Table 5 shows the association between lifestyles and MS onset based on the Japanese criteria with uni- and multi-variable analyses. In the multivariable analysis, smokers showed a significant adjusted HR of MS onset for both men and women in all age groups except for men in their 20s, and men ex-smokers in their 40s. Men with a lower alcohol intake (less than 20 g per day) had a low HR of MS onset in all age groups, and those with a higher alcohol intake (more than 60 g per day) had a high HR in their 30s and 40s. Men with no exercise habit in their 40s, and those with inadequate physical activity in all age groups, and women showing inadequate physical activity in their 40s, had a high HR. Men with a general or fast eating speed in all age groups and women with a fast eating speed in their 30s and 40s had a higher HR than those with slower eating speed. Women who ate dinner less than 2 hours before sleeping, those skipping breakfast, and those with poor sleep in their 40s, and men eating snacks after dinner in their 40s had a high HR.

**Table 5.** Associations between MS and risk factors among age groups during 6-year follow-up

Lifestyles^a^	Women						Men					
	
	20–29 years		30–39 years		40–49 years		20–29 years		30–39 years		40–49 years	
					
	HR	95% CI	HR	95% CI	HR	95% CI	HR	95% CI	HR	95% CI	HR	95% CI
*Univariate analysis^b^*												
Smoking condition												
Non-smoker	1.00		1.00		1.00		1.00		1.00		1.00	
Current smoker	**3.67**	**1.59–8.49**	**2.05**	**1.42–2.98**	**1.74**	**1.36–2.23**	**1.10**	**0.93–1.29**	**1.33**	**1.22–1.45**	**1.31**	**1.21–1.42**
Ex-smoker	1.50	0.34–6.61	0.72	0.39–1.32	**1.44**	**1.07–1.93**	0.82	0.62–1.10	1.03	0.92–1.14	**1.19**	**1.08–1.30**
Alcohol intake												
No drinking	1.00		1.00		1.00		1.00		1.00		1.00	
Less than 20 g per day	0.75	0.25–2.29	**0.61**	**0.38–0.99**	0.95	0.72–1.25	**0.66**	**0.51–0.84**	**0.77**	**0.68–0.87**	**0.84**	**0.76–0.93**
20–60 g per day	1.24	0.43–3.64	1.12	0.74–1.70	**1.35**	**1.04–1.77**	0.94	0.76–1.17	1.05	0.95–1.17	1.08	0.99–1.18
More than 60 g per day	2.41	0.47–12.45	**2.40**	**1.16–4.98**	**1.91**	**1.07–3.40**	1.12	0.81–1.55	**1.31**	**1.13–1.53**	**1.34**	**1.17–1.53**
30 minutes of exercise a week at least twice per week, No	0.99	0.23–4.20	1.59	0.88–2.87	1.32	0.95–1.84	**1.30**	**1.05–1.61**	**1.16**	**1.06–1.28**	**1.20**	**1.11–1.30**
Physical activity over hour per day, No	0.93	0.42–2.06	1.19	0.81–1.75	**1.38**	**1.09–1.74**	**1.23**	**1.05–1.44**	**1.15**	**1.06–0.24**	**1.19**	**1.11–1.27**
Eating speed												
Slow	1.00		1.00		1.00		1.00		1.00		1.00	
General	5.60	0.79–45.42	1.62	0.87–2.98	1.31	0.87–1.96	**1.68**	**1.22–2.33**	**1.52**	**1.29–1.80**	**1.44**	**1.25–1.66**
Fast	7.31	0.92–57.68	**2.43**	**1.29–4.57**	**2.09**	**1.38–3.16**	**2.53**	**1.83–3.48**	**2.31**	**1.95–2.73**	2.06	0.79–2.38
Eating dinner less than 2 hours before sleeping more than 3 times per week	0.84	0.36–1.95	1.09	0.76–1.57	**1.76**	**1.42–2.18**	0.92	0.79–1.08	**1.15**	**1.06–1.23**	**1.08**	**1.02–1.15**
Eating snacks after dinner more than 3 times per week	0.77	0.26–2.24	1.26	0.82–1.92	1.07	0.81–1.41	0.95	0.78–1.16	1.01	0.91–1.11	**1.19**	**1.08–1.29**
Skipping breakfast more than 3 times per week	0.64	0.27–1.61	**1.43**	**1.00–2.05**	**1.69**	**1.34–2.13**	**1.22**	**1.04–1.43**	**1.09**	**1.01–1.18**	**1.06**	0.99–1.14
Sleeping well, No	0.78	0.35–1.72	**1.26**	**0.89–1.78**	**1.55**	**1.24–1.93**	**1.06**	**0.91–1.25**	**1.10**	**1.02–1.19**	**1.08**	**1.02–1.15**

*Multivariable analysis^c^*												
Smoking condition												
Non-smoker	1.00		1.00		1.00		1.00		1.00		1.00	
Current smoker	**3.54**	**1.47–8.53**	**1.70**	**1.15–2.51**	**1.47**	**1.13–1.90**	0.99	0.83–1.18	**1.24**	**1.13–1.35**	**1.23**	**1.13–1.33**
Ex-smoker	1.25	0.28–5.65	0.66	0.35–1.21	1.30	0.96–1.76	0.76	0.57–1.02	0.96	0.86–1.07	**1.12**	**1.02–1.23**
Alcohol intake												
No drinking	1.00		1.00		1.00		1.00		1.00		1.00	
Less than 20 g per day	0.74	0.24–2.28	0.63	0.39–1.01	0.90	0.68–1.20	**0.67**	**0.52–0.86**	**0.78**	**0.69–0.87**	**0.85**	**0.77–0.95**
20–60 g per day	1.10	0.37–3.29	1.09	0.71–1.67	1.13	0.86–1.49	0.91	0.72–1.13	1.01	0.91–1.12	1.08	0.98–1.18
More than 60 g per day	1.80	0.33–9.69	1.92	0.91–4.04	1.30	0.72–2.35	1.07	0.77–1.49	**1.22**	**1.05–1.43**	**1.30**	**1.14–1.48**
30 minutes of exercise a week at least twice per week, No	1.12	0.25–4.95	1.44	0.79–1.64	1.20	0.86–1.69	1.24	0.99–1.55	1.10	0.99–1.22	**1.16**	**1.07–1.27**
Physical activity over hour per day, No	0.96	0.42–2.19	1.11	0.75–1.64	**1.33**	**1.05–1.69**	**1.20**	**1.02–1.41**	**1.13**	**1.04–1.22**	**1.16**	**1.08–1.24**
Eating speed												
Slow	1.00		1.00		1.00		1.00		1.00		1.00	
General	5.21	0.69–39.52	1.57	0.85–2.90	1.35	0.90–2.03	**1.70**	**1.23–2.34**	**1.48**	**1.25–1.75**	**1.45**	**1.26–1.67**
Fast	6.29	0.79–50.01	**2.33**	**1.23–4.39**	**2.04**	**1.35–3.09**	**2.52**	**1.82–3.47**	**2.23**	**1.88–2.64**	**2.07**	**1.79–2.38**
Eating dinner less than 2 hours before sleeping more than 3 times per week	0.92	0.38–2.20	0.93	0.76–1.57	**1.47**	**1.17–1.85**	0.87	0.74–1.03	1.08	0.99–1.16	1.02	0.85–1.09
Eating snacks after dinner more than 3 times per week	0.88	0.30–2.59	1.21	0.78–1.86	0.96	0.72–1.28	0.96	0.78–1.17	1.00	0.91–1.11	**1.18**	**1.08–1.28**
Skipping breakfast more than 3 times per week	0.50	0.19–1.30	1.21	0.83–1.77	**1.38**	**1.08–1.75**	1.17	0.99–1.37	0.99	0.92–1.07	0.97	0.90–1.04
Sleeping well, No	0.81	0.37–1.82	1.14	0.80–1.62	**1.36**	**1.09–1.70**	1.05	0.90–1.24	1.06	0.98–1.14	1.06	0.99–1.12

CI, confidence interval; HR, hazard ratio; MS, metabolic syndrome.

^a^Missing values were excluded in smoking (*n* = 3), alcohol intake (*n* = 40), exercise (*n* = 6), physical activity (*n* = 9), eating speed (*n* = 6), eating dinner less than 2 hours (*n* = 7), eating snacks (*n* = 4), skipping breakfast (*n* = 5), and sleeping (*n* = 6) in women, and smoking (*n* = 1), alcohol intake (*n* = 72), exercise (*n* = 8), physical activity (*n* = 9), eating speed (*n* = 4), eating dinner less than 2 hours (*n* = 8), eating snacks (*n* = 9), skipping breakfast (*n* = 13), and sleeping (*n* = 6) in men.

^b^Using a Cox proportional hazard model for each lifestyle.

^c^Using a Cox proportional hazard model adjusted for age and all of lifestyles.

## DISCUSSION

In the present study, the incidences of MS per 1,000 person-years were 2.2 (adjusted HR 0.19) for women in their 20s and 5.5 (adjusted HR 0.50) for women in their 30s compared with women in their 40s; and 26.3 (adjusted HR 0.46) for men in their 20s and 40.5 (adjusted HR 0.70) for men in their 30s, compared with those in their 40s, during the 6-year follow-up according to the Japanese criteria. To our knowledge, this is the first study to clarify the MS incidence in young Japanese adults in their 20s and 30s by gender.

We also examined the incidence of MS in the young Japanese adults according to NCEP-ATP III criteria. For women, the incidences of MS per 1,000 person-years (6.1 for women in their 20s and 12.5 for women in their 30s) and the adjusted HR of MS (0.29 for women in their 20s and 0.61 for women in their 30s) were higher than those using the Japanese criteria. Conversely, for men, using the NCEP-ATP III criteria, the incidences of MS per 1,000 person-years (12.4 for men in their 20s and 21.2 for men in their 30s) and adjusted HR of MS (0.36 for men in their 20s and 0.64 for men in their 30s) were lower compared with the Japanese criteria. The reason for the differences in the incidence of MS for women and men between the Japanese criteria and NCEP-ATP III was because the WC criterion in NCEP-ATP III was a value of 88 cm and 102 cm or more for men and women, and so the incidence of MS defined using NCEP-ATP III is lower in the men and higher in women than that defined using Japanese criteria. Regarding whether the WC (85 cm or more for men and 90 cm or more for women) in Japanese criteria is an appropriate cut-off point, some studies suggested that the WC was appropriate.^23^^–^^26^

With the NCEP-ATP III criteria, some previous studies in the United States reported that the incidence of MS per 1,000 person-years ranged from 12.0 to 27.9 for young women, and from 20.4 to 24.9 for young men. Compared with those studies, the incidences of MS in young Japanese adults are lower according to our findings, at 10.2 for women and 18.7 for men in the combined 20s and 30s. These results are consistent with epidemiologic statistics showing differences in obesity, diabetes, hypertension, and dyslipidemia between Americans and Japanese.^27^^,^^28^

Furthermore, we explored the relationships between MS onset and age groups in healthy people without any components of MS in the present study. The incidence of MS per 1,000 person-years was much lower. Although women in their 20s and 30s with Japanese criteria and both women and men in their 30s with NCEP-ATP III did not show adjusted HR significantly different from those in their 40s, the *P*-value for trend test in the three groups showed significantly for women and men with both MS criteria. These findings suggest that the incidence of MS per 1,000 person-years for healthy people without any component of MS may gradually increase also with aging.

In addition to aging, a poor lifestyle is an important factor that increases the incidence of MS. In the present study, MS onset was associated with smoking in women in their 20s and 30s, and eating speed in women in their 30s, associated with physical activity, eating speed, and alcohol intake in men in their 20s and 30s, and smoking in men in their 40s. Many previous studies reported that MS onset is associated with smoking,^29^ eating speed,^30^ alcohol intake,^31^ and physical activity.^32^ Other poor lifestyle factors are not significantly correlated with MS onset in young adults, but the impact of each poor lifestyle could not be ignored. When these poor lifestyle factors are combined, their impact on MS onset will be marked, as noted in a previous paper.^33^

Our findings suggest that those in their 20s and 30s had about 20–70% reduced HR of MS defined by both criteria compared with those in their 40s. Prior studies showed that, just as in the middle-aged, young adults with MS show increased rates of all-cause mortality, including IHD and CVD.^26^^,^^34^ Although current specific health checkups and guidance do not include those in their 20s and 30s, the national health program should consider expanding the target age to the 20s and 30s. This would help prevent the onset of MS much earlier.

Strengths of the present investigation are that it involved a large young population and a 6-year cohort, which supports the causative role of MS incidence in the young. Also, the study used two diagnostic criteria for MS, Japanese criteria and NCEP-ATP III criteria, to avoid diagnostic bias caused by different criteria for MS. These are more likely to improve the generalizability of the findings. Finally, our study analyzed the association between age groups and the incidence of MS in healthy people without any components of MS.

Limitations of our study should also be noted. First, about 15,000 (17%) participants who had undergone only one heath check-up at the baseline could not be followed-up. However, there was little withdrawal bias in the present large-scale study. The examinees could freely select their medical examination institution in Japan, Second, the follow-up period was relatively short for healthy people without any components of MS. However, MS onset in men using the Japanese criteria or NCEP-ATP III criteria showed a significant result. If we could extend the follow-up time, there might differences may be become clearer for both Japanese and NCEP-ATP III criteria in women.

In conclusion, the incidences of MS in the 20s and 30s are lower than among those in their 40s, but younger onset accounts for 20–50% of MS incidence for women and 50–70% of MS incidence for men. Our findings suggest that MS in younger people does not look too trivial to be ignored for the population. Thus, early lifestyle intervention for MS is necessary in young adults.
